# Hepatoprotective and renoprotective effects of silymarin against salinomycin-induced toxicity in adult rabbits

**DOI:** 10.14202/vetworld.2022.2244-2252

**Published:** 2022-09-19

**Authors:** Ahmed H. Ghonaim, Mai G. Hopo, Ayman K. Ismail, Tarek R. AboElnaga, Rania Abdelrahman Elgawish, Rania H. Abdou, Kawther A. Elhady

**Affiliations:** 1 Department of Animal Health, Desert Research Center, Cairo, 11435, Egypt; 2 Metra Medical Lab, Ismailia, 41522, Egypt; 3 Department of Forensic Medicine and Toxicology, Faculty of Veterinary Medicine, Suez Canal University, Ismailia, 41522, Egypt; 4 Department of Clinical Pharmacology and Toxicology, College of Medicine, Taif University, Taif, Saudi Arabia

**Keywords:** anticoccidial drugs, feed additives, liver enzymes, oxidative biomarkers, rabbits, salinomycin, silymarin

## Abstract

**Background and Aim:**

Salinomycin sodium, a licensed coccidiostat in rabbits, is used for fattening at a dose of 20–25 mg/kg. Salinomycin toxicity may arise from many risk factors (e.g., overdosage or use in non-target animal species). Silymarin extracted from milk thistle has antioxidant, anti-inflammatory, and antiviral properties. This study aimed to investigate the adverse impacts of oral administration of salinomycin for 28 consecutive days and how to reduce its risks and side effects by administering silymarin.

**Materials and Methods:**

Eighty-four male New Zealand White bucks (1.750–2.000 kg) were randomly divided into seven groups (12 each). Group one was the control. Groups two and three were administered salinomycin orally (doses of 20 and 40 mg/kg ration). Group four was administered salinomycin (20 mg/kg ration) and silymarin (6.5 mg/kg body weight [BW]). Group five received salinomycin (40 mg/kg ration) and silymarin (13 mg/kg BW). Groups six and seven were administered silymarin at doses of 6.5 and 13 mg/kg BW. Rabbits were euthanized and slaughtered on day 29 using the Halal method. Alanine aminotransferase (ALT), aspartate aminotransferase (AST), creatinine, urea, total proteins, albumin, total cholesterol, and high- and low-density lipoprotein (HDL and LDL) were analyzed in serum. Glutathione (GSH), superoxide dismutase (SOD), catalase, and malondialdehyde (MDA) were estimated in the liver. A histopathological investigation was performed on the liver and kidney.

**Results:**

The MDA activity, AST, ALT, total protein, albumin, total cholesterol, triglyceride, LDL, urea, and creatinine values were significantly elevated in groups two and three. The GSH, catalase, SOD, and HDL were significantly lower in these groups than in the control group. There were moderate pathologic changes in the liver and kidney of the third group. However, the results of the fourth and fifth groups improved more than those of the second and third groups. The results of the sixth and seventh groups were nearly the same as those of the control group.

**Conclusion:**

Salinomycin toxicity was caused by oxidative damage because of reactive oxygen species formation. Silymarin (6.5 or 13 mg/kg BW) tends to prevent and treat accidental toxicity. However, the high dose of silymarin (13 mg/kg BW) had more renal and hepatoprotective capacities.

## Introduction

Feed additives are products used in animal nutrition to enhance the feed and the food quality of animal origin or to enhance the health and performance of the animal (e.g., providing upgraded digestibility of the feed materials) [1]. Moreover, feed additives may not be put on the market unless authorized by a scientific assessment, demonstrating that the additive has no harmful effects on human or animal health and the environment. Feed additives are extensively added to animal feed for many purposes, such as anticoccidial drugs and growth promoters [1]. The intensive modern husbandry routines have increased the rate of coccidiosis, consequently causing significant economic losses in poultry production. At present, using anticoccidial feed additives are supposed to be the most effective way to control this disease [2]. In the European Union, 11 coccidiostats are authorized as feed additives for poultry and rabbits. Each anticoccidial was assessed for its target species, applied doses, and administration conditions, including withdrawal times [3]. Among authorized anticoccidial feed additives, ionophore antibiotics (salinomycin, lasalocid, maduramicin, monensin, narasin, and semduramicin) play essential roles. They are used most frequently because of their low cost and high efficiency. The therapeutic index of these coccidiostats is significantly low, and even the target species may be intoxicated when exposed to high levels of those compounds.

Salinomycin sodium is authorized as a coccidiostat to be used in feeds for chickens reared for fattening, with a minimum-maximum concentration of 50–70 mg/kg feed and a withdrawal period of 1 day [4]. Salinomycin sodium is also approved for chickens reared for egg laying at 50 mg/kg feed, without a withdrawal period [4] and for rabbits reared for fattening at 20–25 mg/kg ration, with a withdrawal period of 5 days [4]. Ionophore antibiotic poisoning [5] is due to reactive oxygen species (ROS) formation. The ROS damages cellular macromolecules leading to lipid peroxidation, nucleic acid, and protein alterations. The ROS formation is considered a pathobiochemical mechanism involved in the initiation or progression of numerous diseases, such as atherosclerosis, ischemic heart diseases, diabetes, and the initiation of carcinogenesis or liver diseases. This can result from one of the following reasons:

- A consequence of faults in the calculation of doses
- Excessive ingestion of ionophores because of inappropriate mixing of the drug with the feed [6]
- Administration of ionophores to more susceptible species [7]
- The use of ionophores in combination with other drugs enhances their effects [8].

Hence, antioxidant supplementation may mitigate salinomycin-induced toxicity. Silymarin is a C25-containing flavonoid mixture, extracted from the *Silybum marianum (milk thistle)* plant, which is of Asteriaceae family and has many other names, including milk thistle, Marian thistle, Mary thistle, Mary’s thistle, Saint Mary’s thistle, Blessed milk thistle, Mediterranean milk thistle, variegated thistle, Cardus marianus, and Scotch thistle [9]. Today’s standardized (according to its silibinin, often called silybin, content) silymarin extract contains approximately 65% to 80% flavonolignans (silybin A and silybin B, isosilybin A, isosilybin B, silychristin and silydianin), with small amounts of flavonoids, and approximately 20% to 35% of fatty acids and polyphenolic compounds possessing a range of metabolic regulatory effects. Silybin was discovered as the first member of a new family of natural compounds called flavonolignans in 1959. Silymarin is a polyphenolic flavonoid, with a wide range of biological and pharmacological influences, including antioxidant activity, enhancement of protein synthesis, cell regeneration, and hepatoprotective activities. The potential benefits of silymarin in the treatment of liver diseases have raised many concerns [9]. The rabbit is a classic animal research model. Rabbit meat plays a significant role in human nutrition. The efficiency of rabbits in producing meat compares favorably with that of most other domesticated animals. It can solve a part of the meat shortage in Egypt, especially during poultry crises, such as the bird flu [10]. Numerous antibiotics have been used to promote the growth of farm animals. These products enhance feed conversion and animal growth and decrease morbidity and mortality from diseases. The average growth enhancement was between 4% and 8%. In addition, feed consumption was boosted by 2–5% [10].

This study aimed to investigate the adverse impacts of subacute exposure to salinomycin orally on liver enzymatic activities, protein and lipid profiles, liver oxidative biomarkers, and the structure of the liver and kidneys of adult rabbits. This study also determined how to reduce the risk and side effects of salinomycin by administering silymarin.

## Materials and Methods

### Ethical approval

This study was carried out in strict agreement with recommendations of the Canadian Council on Animal Care guidelines and the protocol was approved by the Ethical Committee of Animal Experiments at Suez Canal University with approval number (2021007).

### Study period and location

The study was conducted from June to September 2020 at the Laboratory animal house, the Faculty of Veterinary Medicine at Suez Canal University.

### Chemicals

Coxistac^®^ (Phibro Animal Health Company, Egypt) was used as a source of salinomycin. Coxistac contains 60 g salinomycin/kg. The dose of salinomycin was calculated based on the daily feed intake of rabbits. Silymarin was available as a Legalon 140 capsule from Cid Pharmaceutical Company, Cairo, Egypt. The dose of silymarin was calculated based on the body weight (BW) of rabbits. Sodium carboxymethylcellulose (CMC) comprises 40–50% cellulose, 25–40% hemicellulose, and 15–35% lignin on a dry basis [11]. The CMC was obtained from Fortune Biotech, China. In this study, CMC was used as a suspending agent to dissolve salinomycin and silymarin, so they could be administered orally to the rabbits.

### Rabbits

Eighty-four adult male New Zealand White bucks (1.750–2.000 kg) were purchased from the International Farm, Ismailia, Egypt. The rabbits were coccidiosis free and kept in individual cages with free access to water. We got the diet from the International Farm in Ismailia and contained no feed additives. The diet composition is shown in Table-1. The daily lighting regime was adjusted to a 10–12 h photoperiod/day. The rabbits were exposed to laboratory conditions for 1 week before the beginning of the experiment.

**Table-1:** Composition of a 22% crude protein creep diet.

Ingredients	Composition %
Oats (ground)	19.0
Wheat (ground)	10.0
Barley (ground)	10.0
Wheat bran	6.4
Soybean meal	12.0
Rapeseed meal	2.5
Fish meal	3.2
Dehydrated alfalfa meal	23.7
Dried Brewer’s yeast	3.0
Dried distillers soluble	4.0
Dried whey	4.0
Molasses	1.0
Salt, iodized	0.5
Vitamin, mineral premix	0.775
DL-methionine	0.07
Feed flavor	0.05

### Experimental design

Rabbits were divided randomly into seven groups (12 rabbits each) and drugs were administered orally through a stomach tube for 28 days. The first group was a control group (received only 1 mL of CMC). The second group was given 20 mg of salinomycin/kg ration dissolved in CMC. The third group was given 40 mg of salinomycin/kg ration dissolved in CMC. The fourth group was given 20 mg of salinomycin/kg ration plus 6.5 mg of silymarin/kg BW dissolved in CMC. The fifth group was given 40 mg of salinomycin/kg BW and 13 mg of silymarin/kg BW dissolved in CMC. The sixth group was given 6.5 mg of silymarin/kg BW dissolved in CMC. The seventh group was given 13 mg of silymarin/kg BW dissolved in CMC. The doses of salinomycin and silymarin were the same as those administered in other studies [4 and 9, respectively], with minor modifications.

### Effects of salinomycin and silymarin on BW gain

The weights of all rabbits in each group were assessed at the beginning of the experiment. Then, there were four successive weekly weight checks. The difference in the weights of rabbits at the beginning of the experiment and after 28 days was calculated.

### Blood sampling

On the 29^th^ experiment day, blood (4 mL) was drawn from the marginal ear vein of the rabbits with the help of a fine 22G needle. Xylene was applied to the target surface to dilate the vein. The blood was allowed to clot before being centrifuged at 1006× *g* for 10 min for biochemical analyses.

### Serobiochemical parameters

Serum was analyzed by spectrophotometer (UV-1800, Shimadzu, Japan) to determine alanine aminotransferase (ALT) and aspartate aminotransferase (AST) levels, according to the study by Wang et al. [12]. Creatinine was determined according to a study by Krishnegowda *et al*. [13]. Urea [14], total proteins [15], albumin [16], total cholesterol [17], and high- and low-density lipoprotein (HDL and LDL) values [18] were estimated by using methods reported previously.

### Effects of salinomycin and silymarin on relative weights of the liver and the kidney

The rabbits were euthanized and slaughtered using the Halal method. The liver and the kidney of the rabbits were immediately dissected and weighed to calculate their relative weights.

### Oxidative stress biomarker assay

The liver was washed with a 0.9% NaCl solution. It was frozen and stored at −20°C for the detection of glutathione (GSH) [19], superoxide dismutase (SOD) [20], catalase [21], and malondialdehyde (MDA) [22]. A commercially available ApoGSH TM Colorimetric Assay Kit from BioVision, USA, was used in these analyses.

### Histopathology

Specimens from the liver and the kidney of different groups were collected and fixed in 10% neutral buffered formalin for 24 h. Subsequently, they were kept in 70% ethanol for histopathological examination. The fixed samples were dehydrated using a series of ethanol graded concentrations, clarified in xylene, embedded in paraffin, and sectioned at 4 μm thickness using a rotary microtome. Hematoxylin and eosin were used to stain the liver sections to examine histological alterations [23].

### Statistical analyses

Data were analyzed (p < 0.05) using Steel and Torrie’s[24] appropriate analysis of variance (ANOVA). A one-way ANOVA in a completely randomized design with four replicates for each trait was considered and followed by Duncan’s as *post hoc* test for pair-wise comparisons. A p ≤ 0.05 was considered statistically significant. All Statistical analyses was performed using the computer program coStat version 6.331[25], to analyze the data.

## Results

### Effects of salinomycin and silymarin on BW gain

The growth rates were significantly affected by different treatments (p < 0.05, while degrees of freedom for groups = 6 and degrees of freedom for replicates = 3). The use of salinomycin (20 mg/kg ration) increased the growth rate of rabbits compared to those in the control group. Salinomycin (40 mg/kg ration) significantly affected weight gain compared with the control group. Silymarin (6.5 mg/kg BW) together with a lower salinomycin dose (20 mg/kg ration) or silymarin (13 mg/kg BW) together with a higher salinomycin dose (40 mg/kg ration) increased the growth rate of rabbits compared to those in the control group. Thus, the use of both silymarin doses (6.5 and 13 mg/kg BW) increased the growth rate of rabbits compared to those in the control group (Table-2).

**Table-2:** Body weight gain (kg) of control and treated rabbits (Mean ± SE).

Group	Initial weight	1^st^ week	2^nd^ week	3^rd^ week	4^th^ week	Weight gain
Control	1.956 ± 0.033	1.978 ± 0.058	2.168 ± 0.047	2.241 ± 0.065	2.316 ± 0.091	0.036 ± 0.0102^a,b^
20 mg SAL/kg ration	2.036 ± 0.095	2.144 ± 0.084	2.381 ± 0.076	2.469 ± 0.068	2.530 ± 0.080	0.494 ± 0.065^a^
40 mg SAL/kg ration	2.053 ± 0.121	2.073 ± 0.107	2.244 ± 0.116	2.390 ± 0.107	2.319 ± 0.106	0.266 ± 0.059^b^
20 mg SAL/kg ration + 6.5 mg SILY/kg BW	2.040 ± 0.034	2.166 ± 0.032	2.420 ± 0.031	2.557 ± 0.043	2.591 ± 0.063	0.551 ± 0.042^a^
40 mg SAL/kg ration + 13 mg SILY/kg BW	2.004 ± 0.058	2.175 ± 0.070	2.358 ± 0.076	2.498 ± 0.070	2.511 ± 0.056	0.507 ± 0.079^a^
6.5 mg SILY/kg BW	1.941 ± 0.102	2.047 ± 0.063	2.150 ± 0.072	2.277 ± 0.071	2.423 ± 0.073	0.481 ± 0.114^a^
13 mg SILY/kg BW	1.934 ± 0.030	1.987 ± 0.044	2.050 ± 0.040	2.243 ± 0.055	2.463 ± 0.026	0.529 ± 0.017^a^

Different letters are significantly different at p *<* 0.05, SAL=Salinomycin, SILY=Silymarin, BW=Body weight, SE=Standard error

### Gross examination

**Figure-1:**
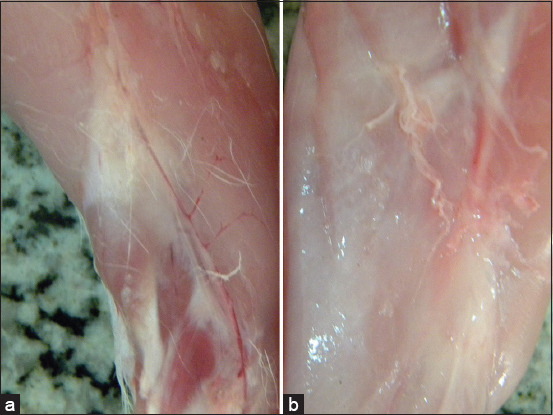
(a) Musculature of control rabbits; (b) musculature of rabbits receiving 40 mg salinomycin/kg ration orally.

No gross lesions were observed in the different organs of rabbits receiving various doses of salinomycin. The exception was that the musculature color of rabbits receiving a 40 mg salinomycin/kg ration was lighter than the musculature of the control group and the other groups, as shown in Figure-1.

### Relative kidney and liver weights

The administration of salinomycin (20 mg/kg ration) did not significantly affect the relative liver and kidney weights (2.74 ± 0.08 and 0.32 ± 0.02, respectively) compared with the control group (2.65 ± 0.06 and 0.29 ± 0.02, respectively). Also, using silymarin (6.5 mg/kg BW) with salinomycin (20 mg/kg ration) maintained the relative liver and kidney weights as in the control group. The administration of salinomycin (40 mg/kg ration) increased the relative liver and kidney weights (3.14 ± 0.04 and 0.34 ± 0.01, respectively) significantly compared to the control group (2.65 ± 0.06 and 0.29 ± 0.02, respectively). However, the administration of silymarin (13 mg/kg BW) with salinomycin (40 mg/kg ration) maintained the relative liver and kidney weights (2.53 ± 0.20 and 0.31 ± 0.01, respectively) as those of the control group (2.65 ± 0.06 and 0.29 ± 0.02, respectively). The use of silymarin (6.5 or 13 mg/kg BW) maintained the relative liver and kidney weights similar to those observed in the control group (Table-3).

**Table-3:** Relative liver and kidney weights of control and treated rabbits (Mean ± SE).

Group	R kidney (g)	Liver (g)
Control	0.29 ± 0.02^b^	2.65 ± 0.06^b^
20 mg SAL/kg ration	0.32 ± 0.02^a,b^	2.74 ± 0.08^b^
40 mg SAL/kg ration	0.34 ± 0.01^a^	3.14 ± 0.04^a^
20 mg SAL/kg ration + 6.5 mg SILY/kg BW	0.28 ± 0.01^b^	2.63 ± 0.05^b^
40 mg SAL/kg ration + 13 mg SILY/kg BW	0.31 ± 0.01^a,b^	2.53 ± 0.20^b^
6.5 mg SILY/kg BW	0.29 ± 0.01^b^	2.56 ± 0.14^b^
13 mg SILY/kg BW	0.30 ± 0.02^b^	2.64 ± 0.09^b^

Different letters are significantly different at p *<* 0.05, SAL=Salinomycin, SILY=Silymarin, BW=Body weight, SE=Standard error

### Liver enzymatic activities

The administration of 20 or 40 mg salinomycin/kg ration resulted in a significant increase in AST and a remarkable increase in ALT activities compared to the control and other treated groups. However, the administration of silymarin (6.5 mg/kg BW) with salinomycin (20 mg/kg ration) or silymarin (13 mg/kg BW) with salinomycin (40 mg/kg ration) maintained AST and ALT values similar to those observed in the control group (Table-4).

**Table-4:** Liver enzymatic activities, total protein, and albumin of control and treated rabbits (Mean ± SE).

Group	AST (U/L)	ALT (U/L)	Total protein (g/dl)	Albumin (g/dl)
Control	8.5 ± 0.87^b^	5.5 ± 0.5^c^	6.8 ± 0.3^a,b,c^	2.0 ± 0.1^a,b^
20 mg SAL/kg ration	10 ± 1.2^a,b^	7.1 ± 0.8^a,b^	6.5 ± 0.4^b,c^	1.7 ± 0.2^b^
40 mg SAL/kg ration	12.3 ± 0.8^a^	8.3 ± 0.6^a^	5.7 ± 0.5^b,c^	1.65 ± 0.15^b^
20 mg SAL/kg ration + 6.5 mg SILY/kg BW	9.3 ± 0.8^b^	5.9 ± 0.4^b, c^	7.9 ± 0.5^a^	2.1 ± 0.1^a^
40 mg SAL/kg ration + 13 mg SILY/kg BW	9.3 ± 0.8^b^	6.3 ± 0.2^b,c^	7.4 ± 0.6^a b^	2.02 ± 0.1^a,b^
6.5 mg SILY/kg BW	8.5 ± 0.9^b^	5.4 ± 0.5^c^	7.0 ± 0.3^a, b,c^	2.2 ± 0.1^a^
13 mg SILY/kg BW	9.3 ± 1.4^b^	5.6 ± 0.2^b, c^	6.9 ± 0.3^a, b,c^	2.2 ± 0.1^a^

Different letters are significantly different at p < 0.05, SAL=Salinomycin, SILY=Silymarin, BW=Body weight, ALT=Alanine aminotransferase, AST=Aspartate aminotransferase, SE=Standard error

### Total protein and albumin

Salinomycin administration (20 or 40 mg/kg in ration) caused a significant decrease in total protein (6.5 ± 0.4 and 5.7 ± 0.5, respectively) and albumin (1.7 ± 0.2 and 1.65 ± 0.15, respectively) compared to the total protein and albumin of the control group (6.8 ± 0.3 and 2.0 ± 0.1, respectively). In contrast, administering low and high doses of silymarin with low and high doses of salinomycin improved the total protein (7.9 ± 0.5 and 7.4 ± 0.6, respectively) and albumin (2.1 ± 0.1 and 2.02 ± 0.1, respectively) levels compared to that of the salinomycin-treated group alone (Table-4).

### Urea and creatinine

Salinomycin (20 or 40 mg/kg ration) significantly increased the urea and creatinine levels compared to the control and other treated groups. Furthermore, silymarin, at either 6.5 or 13 mg/kg BW, with both doses of salinomycin, improved the urea and creatinine values (Table-5).

**Table-5:** Urea and creatinine of control and treated rabbits (Mean ± SE).

Group	Urea	Creatinine
Control	73.4 ± 1.8^d,e^	4.2 ± 0.03^b^
20 mg SAL/kg ration	81.1 ± 1.03^b^	5.1 ± 0.4^a,b^
40 mg SAL/kg ration	94.4 ± 1.5^a^	5.5 ± 0.3^a^
20 mg SAL/kg ration + 6.5 mg SILY/kg BW	77.02 ± 1.6^b,c,d^	4.7 ± 0.2^a,b,c^
40 mg SAL/kg ration + 13 mg SILY/kg BW	78.2 ± 1.7^b,c^	4.3 ± 0.3^c^
6.5 mg SILY/kg BW	76.6 ± 1.3^c,d^	4.5 ± 0.2^b,c^
13 mg SILY/kg BW	72.8 ± 0.8^d,e^	4.2 ± 0.4^b^

Different letters are significantly different at p *<* 0.05, SAL=Salinomycin, SILY=Silymarin, BW=Body weight, SE=Standard error

### Lipid profile

Salinomycin administration (20 or 40 mg/kg ration) induced a significant increase in cholesterol, triglycerides, and LDL, with a considerable decline in HDL compared to the control and other treated groups. The administration of silymarin (6.5 or 13 mg/kg BW) with salinomycin (20 or 40 mg/kg ration) improved cholesterol, triglyceride, LDL, and HDL levels (Table-6).

**Table-6:** Cholesterol, triglycerides, HDL, and LDL of control and treated rabbits (Mean ± SE).

Group	Cholesterol (mg/dl)	Triglycerides (mg/dl)	HDL (mg/dl)	LDL (mg/dl)
Control	138.9 ± 1.1^e^	31.1 ± 0.4^c^	14.3 ± 0.7^d,e^	118.4 ± 1.2^c^
20 mg SAL/kg ration	155.6 ± 1.4^c^	33.2 ± 0.5^b^	12.8 ± 0.4^e,f^	136.1 ± 0.5^b^
40 mg SAL/kg ration	172.2 ± 1.2^a^	35.8 ± 0.8^a^	11.4 ± 0.6^f^	153.7 ± 1.3^a^
20 mg SAL/kg ration + 6.5 mg SILY/kg BW	144.4 ± 1.6^d^	31.6 ± 0.4^b,c^	17.1 ± 0.8^b,c^	121.02 ± 1.1^c^
40 mg SAL/kg ration + 13 mg SILY/kg BW	161.1 ± 0.7^b^	31.6 ± 0.7^b,c^	18.5 ± 0.5^b^	136.3 ± 0.8^b^
6.5 mg SILY/kg BW	133.3 ± 0.9^f^	31.6 ± 0.8^b,c^	15.7 ± 0.5^c,d^	111.3 ± 0.8^d^
13 mg SILY/kg BW	127.03 ± 1.2^g^	30.0 ± 0.7^c^	25.7 ± 0.8^a^	96.1 ± 1.4^e^

Different letters are significantly different at p *<* 0.05, SAL=Salinomycin, SILY=Silymarin, BW: Body weight, HDL=High-density lipoprotein, LDL=Low-density lipoprotein, SE=Standard error

### Oxidative stress biomarkers

Salinomycin (20 or 40 mg/kg in ration) significantly increased MDA activity and decreased GSH, SOD, and catalase activities compared with the control group. When silymarin (6.5 mg/kg BW) was administered in combination with salinomycin (20 mg/kg ration), there was an increase in GSH, SOD, and catalase activities and a decrease in the MDA value compared to those of the salinomycin-treated group alone. Oral silymarin administration (13 mg/kg BW) with salinomycin (40 mg/kg in ration) for 28 successive days resulted in improved GSH, SOD, and catalase activity values and decreased MDA values compared with that of the salinomycin-treated group alone (40 mg/kg ration) (Table-7).

**Table-7:** Oxidative stress biomarkers in rabbits of control and different treated groups (Mean ± SE).

Group	GSH	SOD	Catalase (U/gram tissue)	MDA (nmol/gram tissue)
Control	37.9 ± 0.4^a, b^	5.2 ± 0.1^a^	6.3 ± 0.3^b^	0.2 ± 0.01^e^
20 mg SAL/kg ration	35.2 ± 0.5^c^	4.3 ± 0.1^b, c^	4.8 ± 0.1^c^	0.3 ± 0.01^b^
40 mg SAL/kg ration	29.7 ± 0.8^d^	4.2 ± 0.1^c^	3.8 ± 0.1^d^	0.4 ± 0.01^a^
20 mg SAL/kg ration + 6.5 mg SILY/kg BW	35.8 ± 0.9^b, c^	4.4 ± 0.1^b^	5.1 ± 0.1^c^	0.2 ± 0.01^d^
40 mg SAL/kg ration + 13 mg SILY/kg BW	34.4 ± 0.8^c^	4.2 ± 0.04^b, c^	6.8 ± 0.1^a^	0.3 ± 0.01^c^
6.5 mg SILY/kg BW	35.8 ± 1.0^b, c^	5.1 ± 0.1^a^	6.7 ± 0.1^a, b^	0.2 ± 0.01^e^
13 mg SILY/kg BW	38.8 ± 1.0^a^	5.2 ± 0.1^a^	6.9 ± 0.1^a^	0.2 ± 0.01^e^

Different letters are significantly different at p *<* 0.05, SAL=Salinomycin, SILY=Silymarin, BW: Body weight, GSH=Glutathione, SOD=Superoxide dismutase, MDA=Malondialdehyde, SE=Standard error

### Histopathological results

**Figure-2:**
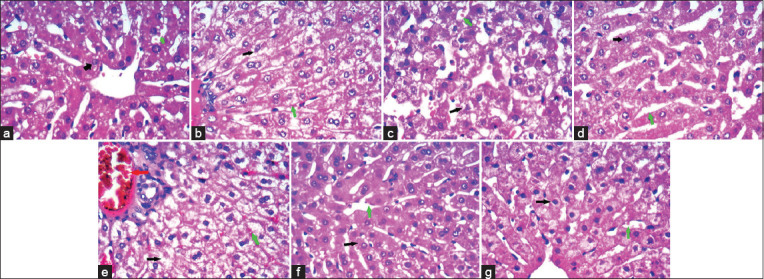
(a) Liver of control rabbits with hepatocytes arranged in hepatic cords and surrounding the central vein (CV) showing abundant cytoplasm and small nuclei (black arrow) arranged in thin cell trabeculae separated by thin wall blood sinusoids (green arrow). (b) Hydropic degeneration with minute foci of coagulative necrosis (black arrow) and normal sinusoids (green arrow) in liver treated with 20 mg salinomycin. (c) Congestion of portal vein with an advanced hydropic swelling (black arrow) and distorted nuclei with congested vessels (red arrowhead) and obliterated sinusoids (green arrow) in liver treated with 40 mg salinomycin. (d) Vacuolated nuclei (glycogen infiltration like) with residual mild hydropic degeneration (black arrow), cell trabeculae in lobular architecture with slightly narrowed sinusoids (green arrow) in liver treated with 20 mg salinomycin and 6.5 mg silymarin. (e) Some mild changes, hepatocytes (black arrow) arranged in thin cell trabeculae in lobular architecture separated by thin wall blood sinusoids (green arrow) in liver treated with 40 mg salinomycin and 13 mg silymarin. (f and g) Hepatocytes (black arrow) showing abundant cytoplasm and small nuclei arranged in thin cell trabeculae separated by thin wall blood sinusoids (green arrow) in liver treated with 6.5 and 13 mg silymarin (H&E stain, 400×).

The liver of rabbits treated with 20 mg salinomycin had hydropic degeneration, with minute foci of coagulative necrosis. In comparison, the liver of rabbits treated with 40 mg salinomycin had a disturbed architecture, portal vein congestion with advanced hydropic swelling, and distorted nuclei. A marked improvement in hepatocytes with thin cell trabeculae in the lobular architecture separated by thin-walled blood sinusoids was observed after using silymarin in both doses administered simultaneously with salinomycin. The liver of individuals in the sixth (6.5 mg silymarin/kg BW) and seventh (13 mg silymarin/kg BW) groups appeared as normal as the liver of individuals in the control group (Figure-2).

**Figure-3:**
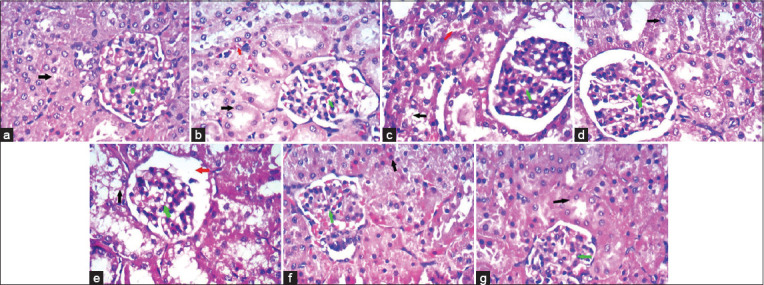
(a) Cloudy swelling of the renal tubular epithelial cells and over condensed glomerular tuft and capsule of control rabbits, glomeruli showed capillary tuft (green arrow), thin Bowman’s space, and mesangial cells, while tubules lined by columnar cells (black arrow) with eosinophilic cytoplasm while interstitium showed blood vessels and stroma. (b) Kidney of rabbits treated with 20 mg salinomycin/kg ration showed dilatation of Bowman’s capsule, star-shaped lumen of BCT, hydropic degeneration (black arrow) of tubular epithelial cells, glomeruli (green arrow) showed a slight increase in mesangial cellularity and stroma congestion (red arrowhead). (c) Kidney of rabbits treated with 40 mg salinomycin/kg ration showed marked glomerular atrophy (green arrow) and hydropic degeneration (black arrow) of tubular epithelial cells with dilatation of Bowman’s space (red arrowhead) with increased mesangial cellularity. (d) Kidney of rabbits treated with 20 mg salinomycin/kg ration + 6.5 mg silymarin/kg BW showed improvement evident as only residual minimal stromal congestion (red arrowhead) and glomerular congestion (green arrow) with regular tubules (black arrow). (e) Kidney of rabbits treated with 40 mg salinomycin/kg ration + 13 mg silymarin/kg BW showed marked improvement evident as regular tubules (black arrow) and glomeruli (green arrow). (f) Kidney of rabbits treated with 6.5 mg silymarin/kg BW formed of glomeruli and tubules, glomeruli showed capillary tuft (green arrow), thin Bowman’s space, and mesangial cells, while tubules lined by columnar cells (black arrow) with eosinophilic cytoplasm while interstitium showed blood vessels and stroma. (g) Kidney of rabbits treated with 13 mg silymarin/kg BW formed of glomeruli and tubules, glomeruli showed capillary tuft (green arrow), thin Bowman’s space, and mesangial cells, while tubules lined by columnar cells (black arrow) with eosinophilic cytoplasm while interstitium showed blood vessels and stroma (H&E stain. 400×).

The kidneys of individuals in the second group (20 mg salinomycin/kg ration) had dilation of the Bowman’s capsule and mild hydropic degeneration of tubular epithelial cells. The kidneys of individuals in the third group (40 mg salinomycin/kg ration) had glomerular atrophy and hydropic degeneration. The kidneys of individuals in the fourth group (20 mg salinomycin/kg ration + 6.5 mg silymarin/kg BW) had improvements that appeared as only residual minimal stromal vascular congestion and glomerular congestion with regular tubules. Moreover, the kidneys of individuals in the fifth group (40 mg salinomycin/kg ration + 13 mg silymarin/kg BW) showed marked improvement, evident in regular tubules and glomeruli. The kidneys of individuals in the sixth (6.5 mg silymarin/kg BW) and seventh (13 mg silymarin/kg BW) groups appeared as normal as the kidneys of individuals in the control group (Figure-3).

## Discussion

This study showed that salinomycin at a dose of 20 mg/kg ration increased the growth rate of rabbits compared to those in the control group. However, salinomycin (40 mg/kg ration) significantly reduced the growth rate of experimental individuals. Salinomycin (40 mg/kg ration) significantly decreased the BW of rabbits from the 3^rd^ week post-treatment. Ghasemi *et al*. [26] reported that this decline in BW might be due to the hazardous influence of salinomycin in the liver, as it is the main organ for protein synthesis. Therefore, it seems that using salinomycin at higher levels reduced protein synthesis, possibly because of oxidative damage to hepatocytes, reduced feed consumption, or even both situations combined.

The gross inspection of the carcasses of different rabbits revealed that the musculature color of rabbits receiving a 40 mg salinomycin/kg ration was lighter than control rabbits. This result agrees with Peixoto *et al*. [27] results, who reported that the musculature of rabbits receiving 100 ppm salinomycin orally for 9 days was generally lighter than normal, with a whitish color similar to that of fish flesh.

Aspartate aminotransferase increased significantly in rabbits administered 40 mg/kg ration of salinomycin. This finding agrees with those of Neufeld [28] who reported increased AST activity with salinomycin at a 50 mg/kg ration and 15.5 g/kg feed, respectively, in Turkeys. However, Bushra *et al*. [29] reported that AST activity in layers was not altered significantly at 60 and 120 ppm salinomycin doses, although it increased significantly at 180 ppm. The ALT activity was markedly increased in the groups receiving 20 and 40 mg/kg of salinomycin in the ration. Elevated levels of AST and ALT might be attributed to oxidative damage caused by free radicals generated by salinomycin, resulting in hepatocellular injury. Moreover, Kamashi *et al*. [30] attributed liver damage to the influence of the toxic dose of salinomycin on hepatocytes and the induction of degenerative changes in the liver. Silymarin supplementation to rabbits alone or in combination with salinomycin improved liver enzymatic activities, which might be due to the antioxidant property of silymarin.

In this study, salinomycin resulted in significant hypoproteinemia with hypoalbuminemia in both doses (20 and 40 mg/kg ration). Kamashi *et al*. [30] found that total proteins were significantly lowered in salinomycin-treated broilers (120 mg/kg feed). However, our results disagreed with those found by Rajaian *et al*. [31], who observed that the total protein concentration was not significantly altered by salinomycin. On the other hand, silymarin supplementation alone or in combination with salinomycin improved the total protein and albumin to normal levels. Histopathological findings of the liver were confirmed by the observed elevation in enzymatic activity and decrease in total protein and albumin in salinomycin-treated rabbits, where there were mild pathological alterations in the liver of rabbits treated with 20 mg/kg of salinomycin in ration and moderate pathological alterations in the liver of rabbits treated with 40 mg/kg of salinomycin in the ration. These results agree with those of several researchers who demonstrated the destructive influence of carboxylic ionophores on the liver [32]. However, the administration of silymarin improved these alterations in the liver because of its hepatoprotective properties. Our results agree with those of many investigators who have mentioned that silymarin has protective effects against lipid peroxidation and histopathological alterations [33].

The blood urea concentration increased significantly in rabbits receiving 20 and 40 mg/kg of salinomycin in the ration. Serum creatinine levels were significantly higher in rabbits given 20 or 40 mg/kg of salinomycin. This follows the results of Kamashi *et al*. [30], who reported elevated serum creatinine and blood urea concentrations in broilers following salinomycin treatment. The apparent increase in creatinine and blood urea values, especially in the salinomycin-treated group, may reveal a reduction in the glomerular filtration rate and impairment of renal blood flow. However, silymarin supplementation alone or in combination with salinomycin improved urea and creatinine levels. Further, our histopathological findings of the kidney confirmed the observed elevations in urea and creatinine, where there were mild pathologic changes in the second group (20 mg/kg of salinomycin in ration) and moderate pathologic changes in the third group (40 mg/kg of salinomycin in ration). These results agree with those of Peixoto *et al*. [27], who reported congestion of the kidneys in rabbits administered 100 ppm salinomycin, and with those of Ashrafihelan *et al*. [32], who reported acute tubular necrosis in adult sheep exposed to 22,388 ppm salinomycin. The administration of silymarin (6.5 mg/kg BW) with salinomycin (20 mg/kg ration) or silymarin (13 mg/kg BW) with salinomycin (40 mg/kg ration) orally for 28 successive days resulted in an improved state of the kidney.

Total cholesterol, triglycerides, and LDL were notably increased, with a significant reduction in HDL in the serum of rabbits receiving 20 and 40 mg/kg of salinomycin in the ration. This could indicate cardiac damage and renal impairment, possibly due to free radical-induced oxidative damage. These results agree with Kamashi *et al*. [30], who reported that the levels of total lipids, triglycerides, total cholesterol, and LDL were significantly increased in broiler chickens fed on food-containing salinomycin (120 mg/kg feed). Abnormal hyperlipidemic conditions may be idiopathic or secondary to hepatic insufficiency and nephrotic syndrome [33]. The administration of silymarin resulted in lower cholesterol, triglyceride, and LDL levels, and higher HDL levels, which could be attributed to the antioxidant activity of silymarin [34].

Oxidative damage results from an imbalance between oxidants and antioxidants at the cellular level. This process involves oxidative alteration of cellular macromolecules, cell death by apoptosis or necrosis, and structural tissue damage [35]. In animals intoxicated with salinomycin, tissue injury and necrosis occur in various organs, particularly in the myocardium and skeletal muscle [36]. Salinomycin (20 mg/kg ration) caused a remarkable increase in MDA activity and a significant decline in GSH, SOD, and catalase activities compared with the control rabbits. When silymarin (6.5 mg/kg BW) was combined with salinomycin, it enhanced the GSH, SOD, and catalase activity values and decreased the MDA value. Our result agrees with those of Hajimohammadi *et al*. [37], who observed a significant decline in SOD, catalase, and glutathione peroxidase (GPX) in salinomycin-treated sheep. Our findings showed that salinomycin intoxication increases the levels of lipid peroxidation products. Miyase *et al*. [38] reported that MDA evaluation is usually used to reveal lipid peroxidation. Similar to our findings, Kargin and Fidanci [39] reported elevated levels of lipid peroxidation (MDA) in numerous diseases, including kidney diseases. Hajimohammadi *et al*. [37] also observed an increase in MDA in salinomycin-treated sheep compared with the control group.

## Conclusion

This study highlighted the risk of using salinomycin in rabbit feed. Hence, it is recommended that salinomycin be used as a growth promoter for rabbits. Nonetheless, its concentration must not exceed 20 mg/kg in the ration of the animals. In addition, it should be homogeneously mixed with the ration. On the other hand, the duration of salinomycin administration must not exceed 30 days. The continued use of silymarin prevented and cured salinomycin-induced toxicity in rabbits, having reversed and resettled its toxic effects and serum biochemical patterns.

## Authors’ Contributions

All authors contributed to the study design, interpreted the data, and wrote the manuscript. AHG: First and corresponding author, data collection, investigation and methodology, data curation, statistical analysis, and drafted the manuscript. MGH, TRA, and RHA: Statistical analysis, drafted and edited the manuscript. RAE, AKI, and KAE: Provided technical help during the experiments and revised the manuscript. All authors have read and approved the final manuscript.

## Data Availability

The datasets generated during and analyzed during the present study are not publicly available due to privacy but are available from the corresponding author on reasonable request.
